# Decompressive laparotomy for abdominal compartment syndrome resulting from severe acute pancreatitis: a case report

**DOI:** 10.1186/s12876-019-1059-0

**Published:** 2019-08-08

**Authors:** Shinya Ikeda, Takuma Kagami, Shinya Tani, Takahiro Uotani, Mihoko Yamade, Yasushi Hamaya, Yoshifumi Morita, Takanori Sakaguchi, Satoshi Osawa, Ken Sugimoto

**Affiliations:** 1 First Department of Medicine, Hamamatsu University School of Medicine, 1-20-1 Handayama, Higashi-ku, Hamamatsu, 431-3192 Japan; 2 Department of Endoscopic and Photodynamic Medicine, Hamamatsu University School of Medicine, Hamamatsu, 431-3192 Japan; 3 Second Department of Surgery, Hamamatsu University School of Medicine, Hamamatsu, 431-3192 Japan

**Keywords:** Abdominal compartment syndrome, Decompressive laparotomy, Surgical abdominal decompression, Acute pancreatitis, Severe acute pancreatitis

## Abstract

**Background:**

Abdominal compartment syndrome (ACS) is associated with mortality in patients with critical illness such as severe acute pancreatitis, but it remains unclear whether decompressive laparotomy for ACS can improve the prognosis of patients.

**Case presentation:**

A woman in her 60s visited our hospital because of upper abdominal pain. On the basis of her laboratory data and abdominal contrast-enhanced computed tomography findings, acute gallstone pancreatitis was diagnosed. She underwent endoscopic sphincterotomy for the removal of the common bile duct stone. Then, a drainage tube was placed in the bile duct. However, on the 5th hospital day, her intra-abdominal pressure increased to 22 mmHg and renal dysfunction was observed, which led to the diagnosis of ACS. As intensive medical treatments did not improve her ACS, she underwent decompressive laparotomy on the 9th hospital day. Postoperatively, her laboratory data and intravesical pressure improved, and she was discharged from the hospital after abdominal closure, continuous drainage, and antibiotic therapy.

**Conclusion:**

As the effectiveness of decompressive laparotomy for ACS has not been established, this treatment indication remains controversial. Decompressive laparotomy is considered useful for the management of ACS, if it is performed at an appropriate time, as in the present case.

## Background

The incidence of abdominal compartment syndrome (ACS) resulting from severe acute pancreatitis has been reported to be 4–27% [1–3]. The mortality is high (50–75%) in patients who have severe acute pancreatitis and develop ACS [3–5]. In severe acute pancreatitis, increased vascular permeability due to excessive inflammation, increased intraperitoneal volume associated with paralytic ileus, and decreased abdominal wall compliance due to edema result in the development of ACS [6, 7]. The goal of the treatment of ACS is to reduce intra-abdominal pressure by removing the contents in the intestinal tract and lesions in the abdominal cavity, improve abdominal wall compliance, optimize fluid administration, and optimize systemic and regional perfusion [8, 9]. If there is no response to these interventions, surgical abdominal decompression should be considered [8, 9].

As the effectiveness of decompressive laparotomy for ACS has not been established, this treatment indication remains controversial. In the present case, we experienced that decompressive laparotomy for ACS resulting from severe acute pancreatitis was very effective.

## Case presentation

A woman in her 60s visited our hospital because of upper abdominal pain. She had a history of appendicitis and cholelithiasis. She was diagnosed with acute gallstone pancreatitis based on her laboratory data and abdominal computed tomography (CT) findings.

**Fig. 1:**
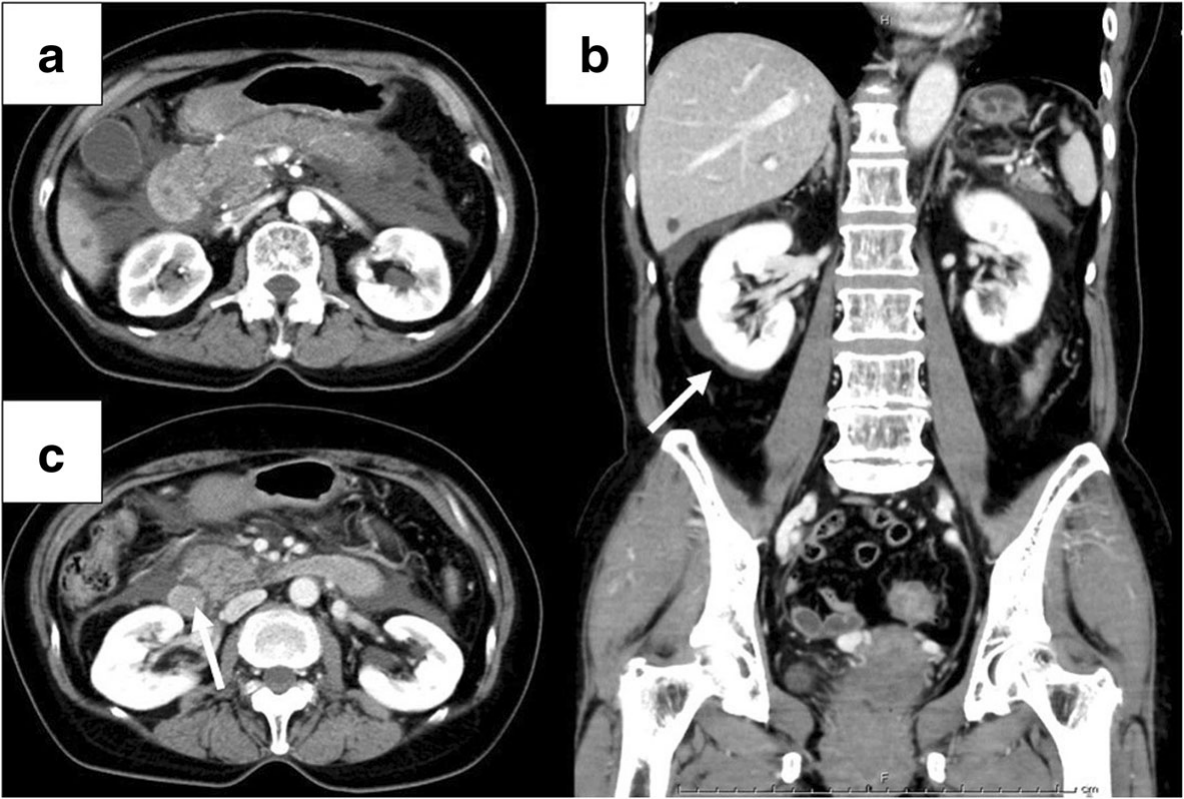
**a** Abdominal contrast-enhanced CT showed pancreas enlargement, especially in the head of the pancreas. The pancreas showed homogenous enhancement. **b** Abdominal contrast-enhanced CT revealed fluid collection around the pancreas spread beyond lower kidney edge (white arrow). **c** There was a common bile duct stone (white arrow)

Her physical examination at admission revealed that her blood pressure was 101/69 mmHg, body temperature was 36.1 °C, pulse rate was 56 beats per minute, oxygen saturation was 97% with room air, and abdomen had epigastric tenderness without muscle guarding or rigidity. Laboratory investigations revealed a white blood cell count of 21.1 × 10^9^/L, hemoglobin level of 14.0 g/dL, and platelet count of 316 × 10^9^/L. Red blood cell count and serum creatinine level were within the normal range. Serum amylase and serum lipase levels were elevated to 2313 U/L and 868 U/L, respectively. Other blood tests showed mild elevations in the biliary enzymes as follows: aspartate aminotransferase 29 U/L, alanine aminotransferase 55 U/L, alkaline phosphatase 654 U/L, γ-glutamyl transpeptidase 168 U/L, and total bilirubin level 0.8 mg/dL. Arterial blood gas analysis was normal. Abdominal contrast-enhanced CT (Fig. 1) showed enlargement of the pancreas, especially in the head of the pancreas, fluid collection around the pancreas, and choledocholithiasis. The pancreas showed homogeneous enhancement.

On the 1st hospital day, the patient was not allowed to eat or drink, and intravenous therapy with an antibacterial drug (doripenem) and a protease inhibitor (ulinastatin) was started. She underwent endoscopic retrograde cholangiopancreatography for common bile duct stone removal. Endoscopic sphincterotomy was performed, and a drainage tube was placed in the bile duct.

**Fig. 2:**
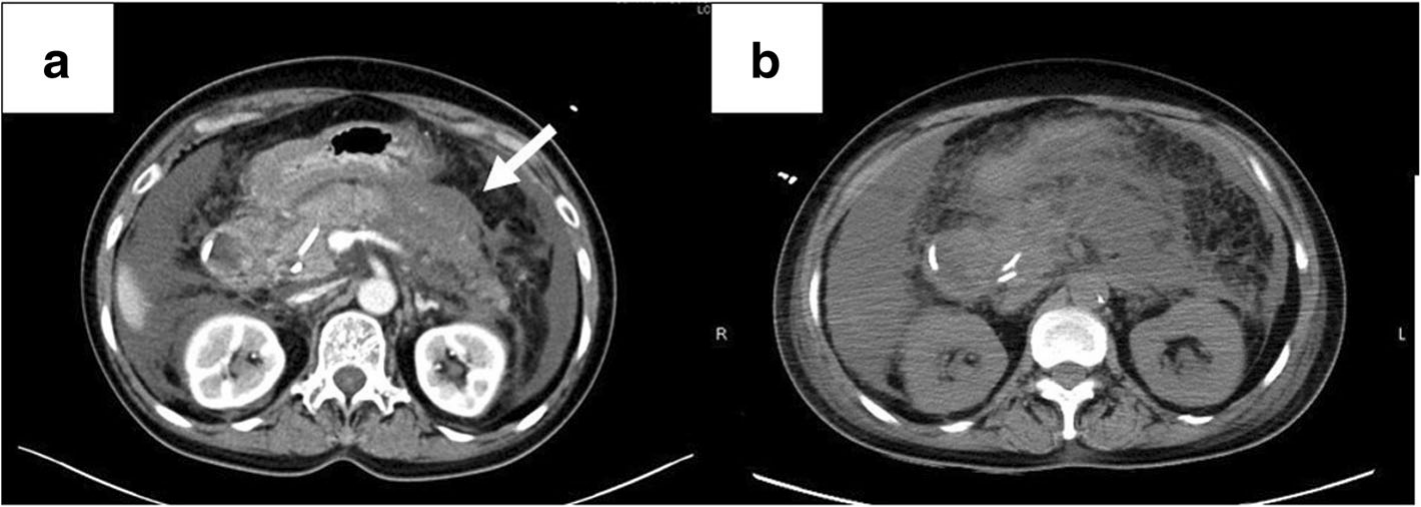
**a** Abdominal contrast-enhanced CT showed contrast failure (white arrow) and extension of surrounding inflammation on the 2nd hospital day. **b** Abdominal CT revealed progression of pancreatic enlargement and paralytic ileus on the 5th hospital day

On the 2nd hospital day, her laboratory data worsened, and abdominal contrast-enhanced CT showed poor contrast of the pancreas, which led to suspected pancreatic necrosis being suspected, and spread of inflammation around the pancreas (Fig. 2a). The patient was diagnosed with severe acute pancreatitis and admitted to the intensive care unit (ICU). She was intubated due to respiratory failure, and a vasopressor was started to reverse circulatory failure on the 4th hospital day.

**Fig. 3:**
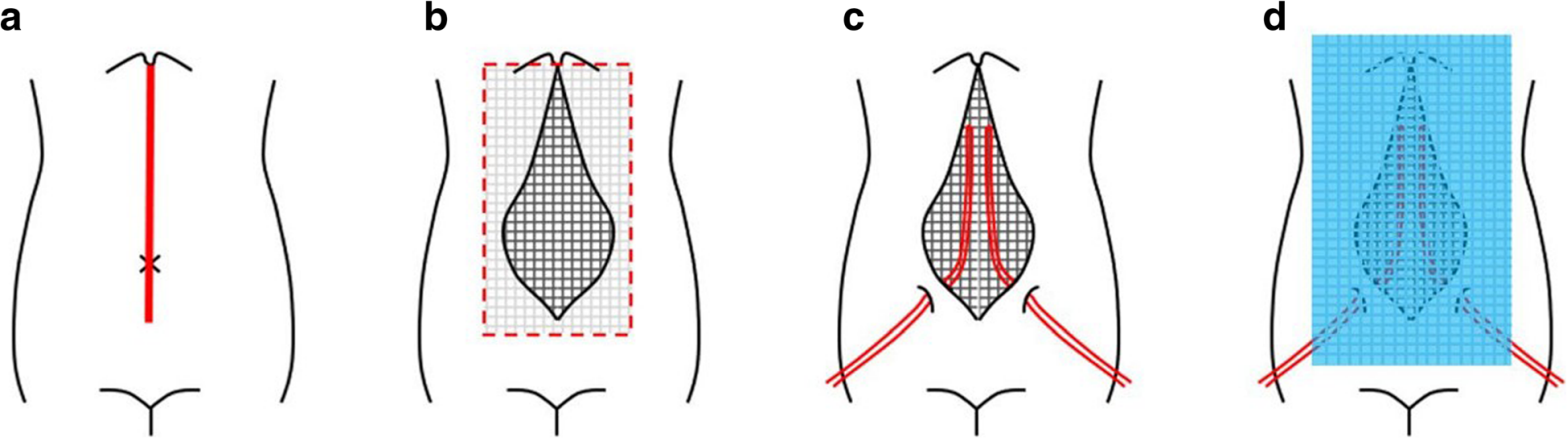
**a** A midline incision was made, and ascites drainage was performed. **b** A gauze was spread in the abdominal cavity. c Two drainage tubes were inserted. 4d A sterile drape was used to cover the wound

**Fig. 4:**
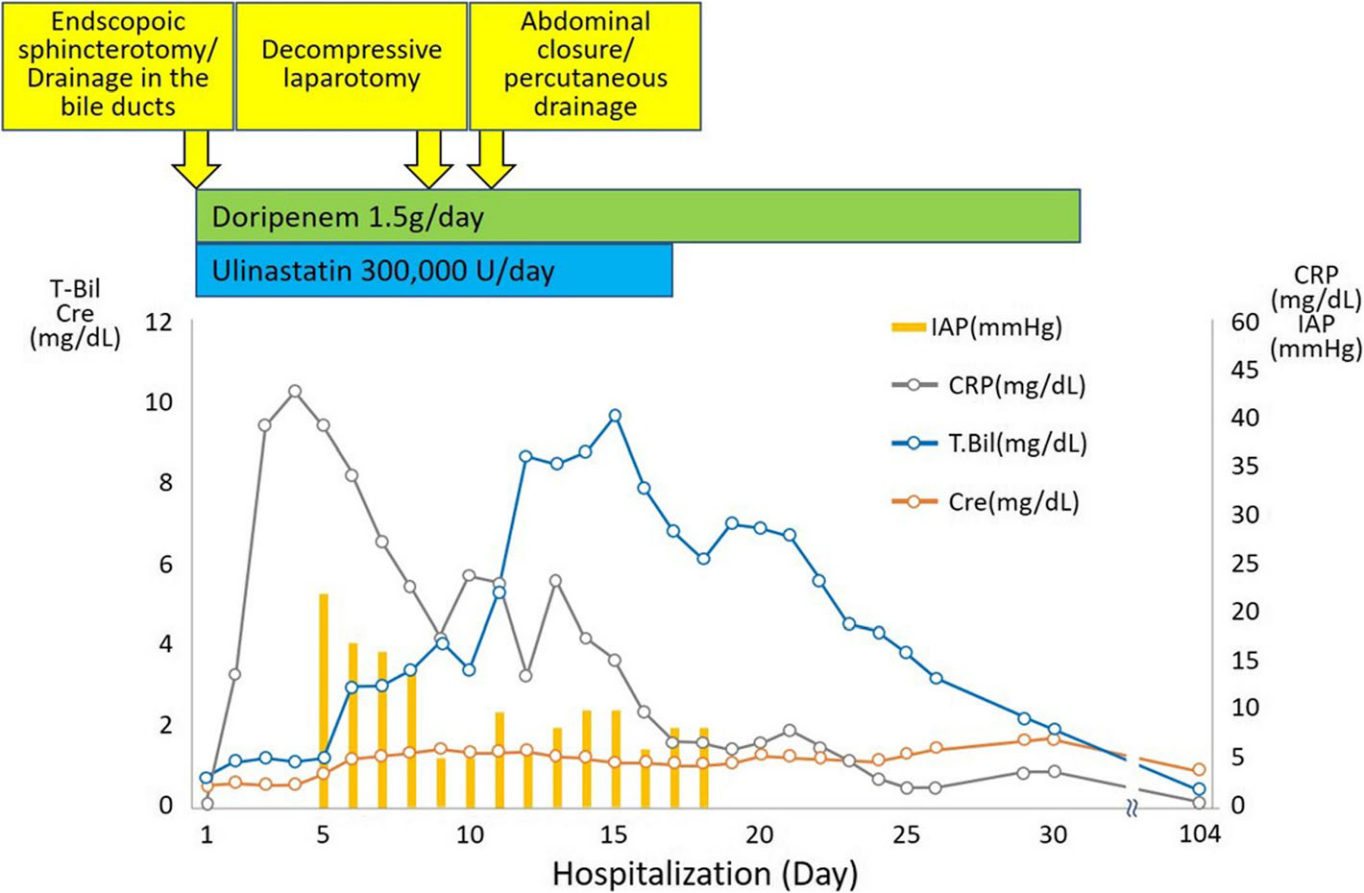
The clinical course of the patient. DRPM: Doripenem, CRP: C-reactive protein, IAP: Intra-abdominal pressure

On the 5th hospital day, the patient showed progressive abdominal distension and decreased bowel sounds. Abdominal CT indicated progressive enlargement of the pancreas and paralytic ileus (Fig. 2b). Her intra-abdominal pressure, measured from her intravesical pressure, increased to 22 mmHg, and renal dysfunction was also observed, leading to the diagnosis of ACS [10, 11]. Gastrointestinal prokinetic agent and neuromuscular blockade were initiated, and a nasogastric tube was inserted to drain the intraluminal contents. As these medical treatments did not improve her ACS, the patient underwent decompressive laparotomy with a midline incision on her 9th hospital day (Fig. 3). Postoperatively, her laboratory data and intra-abdominal pressure improved. However, despite administration of the antibiotic, the white blood cell count and C-reactive protein level deteriorated, and the necrotizing pancreatitis also did not improve. Therefore, when abdominal closure was performed on her 11th hospital day, percutaneous drainage tubes around the pancreas were inserted simultaneously. Continuous drainage and antibiotic therapy resulted in improvement of necrotizing pancreatitis, and she was discharged on the 104th hospital day. The drainage tubes were removed seven days after her discharge. The clinical course of this patient is shown in Fig. 4.

## Discussion and conclusions

ACS is defined as the state where the abdominal pressure is 20 mmHg or higher, which leads to various clinical symptoms such as respiratory insufficiency, circulatory insufficiency, and organ failure [10, 11].

ACS generally occurs in critically ill patients who have burns or traumatic injuries or have undergone transplant surgery [12]. ACS is of two types, namely primary and secondary [10]. Primary ACS is caused by injury or disease in the abdominal pelvic area, such as abdominal trauma and pancreatitis, often requiring surgical or radiological intervention [10]. In contrast, secondary ACS occurs due to factors other than injury or disease in the abdominal or pelvic area, such as burns, sepsis, and fluid resuscitation [10].

ACS has been reported to increase mortality in patients with severe acute pancreatitis. Recently, the mortality rate has been shown to be 50–75% in patients who developed ACS after severe acute pancreatitis and 11% in patients who did not have ACS [3–5, 13].

Intra-abdominal pressure can be measured indirectly by using, for example, an intragastric or intravesical catheter [14]. This method allows for a minimally invasive, easy, and accurate measurement of intravesical pressure; hence, it is a standard method for monitoring intra-abdominal pressure and diagnosing ACS [15]. As intravesical pressure fluctuates depending on the position of the head, the positions of the head and body must be matched in every measurement [15–17]. On the basis of the risk assessment for intra-abdominal hypertension, routine monitoring of intra-abdominal pressure has been advocated to enable early diagnosis in various conditions other than emergency surgical operation and external wound and in critically ill patients with overt ACS. Once intra-abdominal hypertension is observed, careful monitoring of the intra-abdominal pressure and organ functions needs to be performed along with appropriate measures to prevent further organ dysfunction and irreversible damage. In our case, it was possible to diagnose ACS early by monitoring the intravesical pressure in the ICU.

Management of ACS involves removing intraabdominal contents and lesions, improving abdominal wall compliance, and optimizing fluid administration and systemic perfusion [8, 9]. Management of ACS includes nasogastric tube insertion, use of gastrointestinal prokinetics, neuromuscular blockade, sedatives, analgesics, renal replacement therapy, and percutaneous drainage [8, 9]. When these treatment modalities fail to relieve ACS, surgical intervention is required [8, 9]. Decompressive laparotomy successfully lowers the intra-abdominal pressure [12]. However, the mortality rate of severe acute pancreatitis with ACS is high (54%) [12].

Currently, no clear guidelines have been established for the indication of surgical intervention for patients with ACS due to severe acute pancreatitis. Further studies are required to clarify which cases are appropriate for surgical intervention. Some reports show that early decompression may improve survival. An analysis of 26 ACS patients with severe acute pancreatitis who underwent surgical decompression at a tertiary care hospital showed a decrease in mortality when surgery was performed within 4 days of disease onset (18% vs. 100%) [18]. In addition, 16 of 45 patients with severe acute pancreatitis in the ICU required surgical decompression, and the average time to surgery was 3.1 h from the diagnosis of ACS [19]. However, no significant difference in mortality was found between the group that required surgery and the group that did not require surgery (24% vs. 25%, *P* = 0.9) [19]. Boone et al. followed up 12 patients with severe acute pancreatitis who underwent decompressive laparotomy for the treatment of ACS over a 9-year period [5]. The decompressive laparotomy was performed within 4.5 days after ACS onset, with significant improvement in several physiological findings [5]. Thus, they concluded that early surgical decompression contributed to the improved prognosis in patients with these fatal complications [5]. These studies indicate that early surgical decompression in patients with ACS during severe acute pancreatitis is associated with a lower mortality rate. In our case, early surgical decompression (four days after being diagnosed with ACS) could be one of the important factors in saving the patient of the patient.

As the effectiveness of decompressive laparotomy for ACS has not been established, the indication of this treatment remains controversial. Decompressive laparotomy is considered useful for the management of ACS if performed at an appropriate time, as in the present case.

## Data Availability

Not applicable.

## Abbreviations

ACS: Abdominal compartment syndrome
CT: Computed tomography
ICU: Intensive care unit

## References

[1] Tao J, Wang C, Chen L, Yang Z, Xu Y, Xiong J (2003). Diagnosis and management of severe acute pancreatitis complicated with abdominal compartment syndrome. J Huazhong Univ Sci Technolog Med Sci.

[2] Jacob AO, Stewart P, Jacob O (2016). Early surgical intervention in severe acute pancreatitis: central Australian experience. ANZ J Surg.

[3] Chen H, Li F, Sun J-B, Jia J-G (2008). Abdominal compartment syndrome in patients with severe acute pancreatitis in early stage. World J Gastroenterol.

[4] Al-Bahrani AZ, Abid GH, Holt A, McCloy RF, Benson J, Eddleston J (2008). Clinical relevance of intra-abdominal hypertension in patients with severe acute pancreatitis. Pancreas..

[5] Boone B, Zureikat HSJ, Moser AJ, Yaday D, Zeh HJ (2013). Abdominal compartment syndrome is an early, lethal complication of acute pancreatitis. Am Surg.

[6] Leppäniemi A, Johansson K, De Waele JJ (2007). Abdominal compartment syndrome and acute pancreatitis. Acta Clin Belg.

[7] De Waele JJ, Leppäniemi AK (2009). Intra-abdominal hypertension in acute pancreatitis. World J Surg.

[8] Kirkpatrick AW, Roberts DJ, De Waele J, Jaeschke R, Malbrain ML, De Keulenaer B (2013). Intra-abdominal hypertension and the abdominal compartment syndrome: updated consensus definitions and clinical practice guidelines from the world Society of the Abdominal Compartment Syndrome. Intensive Care Med.

[9] Smit M, Buddingh KT, Bosma B, Nieuwenhuijs VB, Hofker HS, Zijlstra JG (2016). Abdominal compartment syndrome and intra-abdominal ischemia in patients with severe acute pancreatitis. World J Surg.

[10] Malbrain ML, Cheatham ML, Kirkpatrick A, Sugrue M, Parr M, De Waele J (2006). Results from the international conference of experts on intra-abdominal hypertension and abdominal compartment syndrome. I. Definitions. Intensive Care Med.

[11] Cheatham ML, Malbrain ML, Kirkpatrick A, Sugrue M, Parr M, De Waele J (2007). Results from the international conference of experts on intra-abdominal hypertension and abdominal compartment syndrome. II. Recommendations. Intensive Care Med.

[12] Malbrain ML, Chiumello D, Pelosi P, Bihari D, Innes R, Ranieri VM (2005). Incidence and prognosis of intraabdominal hypertension in a mixed population of critically ill patients: a multiple-center epidemiological study. Crit Care Med.

[13] Malbrain ML, Chiumello D, Pelosi P, Wilmer A, Brienza N, Malcangi V (2004). Prevalence of intra-abdominal hypertension in critically ill patients: a multicentre epidemiological study. Intensive Care Med.

[14] van Brunschot S, Schut AJ, Bouwense SA, Besselink MG, Bakker OJ, van Goor H (2014). Abdominal compartment syndrome in acute pancreatitis: a systematic review. Pancreas.

[15] Malbrain ML (2004). Different techniques to measure intra-abdominal pressure (IAP): time for a critical re-appraisal. Intensive Care Med.

[16] Cheatham ML, De Waele JJ, De Laet I, De Keulenaer B, Widder S, Kirkpatrick AW (2009). The impact of body position on intra-abdominal pressure measurement: a multicenter analysis. Crit Care Med.

[17] De Keulenaer BL, De Waele JJ, Powell B, Malbrain ML (2009). What is normal intra-abdominal pressure and how is it affected by positioning, body mass and positive end-expiratory pressure?. Intensive Care Med.

[18] Mentula P, Hienonen P, Kemppainen E, Puolakkainen P, Leppäniemi A (2010). Surgical decompression for abdominal compartment syndrome in severe acute pancreatitis. Arch Surg.

[19] Davis PJ, Eltawil KM, Abu-Wasel B, Walsh MJ, Topp T, Molinari M (2013). Effect of obesity and decompressive laparotomy on mortality in acute pancreatitis requiring intensive care unit admission. World J Surg.

